# Chronic and Transient Hyperglycemia Induces Changes in the Expression Patterns of *IL6* and *ADIPOQ* Genes and Their Associated Epigenetic Modifications in Differentiating Human Visceral Adipocytes

**DOI:** 10.3390/ijms22136964

**Published:** 2021-06-28

**Authors:** Adam Wróblewski, Justyna Strycharz, Ewa Świderska, Aneta Balcerczyk, Janusz Szemraj, Józef Drzewoski, Agnieszka Śliwińska

**Affiliations:** 1 Department of Medical Biochemistry, Medical University of Lodz, 92-215 Lodz, Poland; justyna.strycharz@umed.lodz.pl (J.S.); ewa.swiderska@umed.lodz.pl (E.Ś.); janusz.szemraj@umed.lodz.pl (J.S.); 2 Department of Molecular Biophysics, University of Lodz, 90-236 Lodz, Poland; aneta.balcerczyk@biol.uni.lodz.pl; 3 Central Teaching Hospital of the Medical University of Lodz, 92-213 Lodz, Poland; jozef.drzewoski@umed.lodz.pl; 4 Department of Nucleic Acids Biochemistry, Medical University of Lodz, 92-213 Lodz, Poland

**Keywords:** visceral preadipocytes, adipocytes, interleukin 6, adiponectin, adipogenesis, histone modifications, microRNA, inflammation, hyperglycemia, diabetes

## Abstract

Adipokines secreted by hypertrophic visceral adipose tissue (VAT) instigate low-grade inflammation, followed by hyperglycemia (HG)-related metabolic disorders. The latter may develop with the participation of epigenetic modifications. Our aim was to assess how HG influences selected epigenetic modifications and the expression of interleukin 6 (IL-6) and adiponectin (APN; gene symbol ADIPOQ) during the adipogenesis of human visceral preadipocytes (HPA-v). Adipocytes (Ads) were chronically or transiently HG-treated during three stages of adipogenesis (proliferation, differentiation, maturation). We measured adipokine mRNA, protein, proven or predicted microRNA expression (RT-qPCR and ELISA), and enrichment of H3K9/14ac, H3K4me3, and H3K9me3 at gene promoter regions (chromatin immunoprecipitation). In chronic HG, we detected different expression patterns of the studied adipokines at the mRNA and protein levels. Chronic and transient HG-induced changes in miRNA (miR-26a-5p, miR-26b-5p, let-7d-5p, let-7e-5p, miR-365a-3p, miR-146a-5p) were mostly convergent to altered IL-6 transcription. Alterations in histone marks at the *IL6* promoter were also in agreement with IL-6 mRNA. The open chromatin marks at the *ADIPOQ* promoter mostly reflected the APN transcription during NG adipogenesis, while, in the differentiation stage, HG-induced changes in all studied marks were in line with APN mRNA levels. In summary, HG dysregulated adipokine expression, promoting inflammation. Epigenetic changes coexisted with altered expression of adipokines, especially for IL-6; therefore, epigenetic marks induced by transient HG may act as epi-memory in Ads. Such changes in the epigenome and expression of adipokines could be instrumental in the development of inflammation and metabolic deregulation of VAT.

## 1. Introduction

Among the various types of adipose tissue (AT), visceral adipose tissue (VAT) prevails in its contribution to the development and progression of metabolic disorders. VAT hypertrophy and hyperplasia, local hypoxia, and subsequent apoptosis of adipocytes (Ads) are factors inducing macrophage infiltration, especially into VAT, which enhances low-grade chronic inflammation, characterized by a detrimental profile of secreted adipocytokines [1,2]. Inflamed, overgrown VAT develops impaired glucose uptake through disrupted insulin signaling [1]. Therefore, visceral obesity may lead to the development of insulin resistance (IR), prediabetes, and eventually type 2 diabetes mellitus (T2DM). Ads are removed by macrophages resident in AT and constantly renewed from adipose stem cells’ reservoir in a complicated multi-stage adipogenesis process [1,2,3]. The first stage of adipogenesis involves the commitment of the mesenchymal stem cells to the adipocyte’s fate, and they change into fibroblast-like preadipocytes. Subsequently, differentiation and maturation occur to form adipocytes that are able to store fat in the form of lipid droplets. The differentiation of preadipocytes involves a transcriptional cascade including peroxisome proliferator-activated receptor-γ (PPAR-γ) and CCAAT-enhancer-binding proteins (C/EBPs), which manage the process of adipogenesis. Finally, maturation gives rise to fully functional Ads capable of accumulating lipids and producing adipokines [3]. Apart from energy storing, AT functions as a complex paracrine and endocrine organ that secretes specific cytokines (i.e., adipokines), e.g., leptin, adiponectin (APN), resistin, visfatin, monocyte chemotactic protein-1, interleukin 6 (IL-6,) and tumor necrosis factor α (TNF-α), and is involved in the energy balance and inflammatory response [4].

IL-6, encoded by the *IL6* gene, is a widespread cytokine, produced mainly in macrophages and, among others, endothelial cells, hepatocytes, myocytes, and adipocytes. Among the various actions of IL-6, which may depend on the target tissue and other cytokines, the most pronounced is the promotion of inflammatory responses [5]. Upon dysregulation, intensively expressed IL-6 (mostly transactivated by the nuclear factor-kappa β (NF-κβ) signaling pathway) leads to low-grade chronic inflammation, which contributes to T2DM or metabolic syndrome (MetS), as IL-6 has been demonstrated to impair insulin signaling in Ads and hepatocytes [1,5]. Moreover, increased plasma IL-6 levels may predict the onset of T2DM, whereas in AT expression of IL-6, mRNA is positively associated with obesity and augmented in IR [5].

APN (encoded by the *ADIPOQ* gene) is an abundantly expressed protein in Ads, serving as a marker of adipogenic differentiation (its former name was apM1—adipose most abundant gene transcript 1). Importantly, APN induces glucose uptake and fatty acid oxidation while attenuating gluconeogenesis and, therefore, enhancing the insulin sensitivity of peripheral tissues. APN serves as an anti-inflammatory adipokine by stimulating the polarization of macrophages into anti-inflammatory M2 macrophages. Moreover, the production of TNF-α in macrophages can be abrogated by APN through NF-κβ inhibition [6]. Inflamed AT secretes lower levels of APN, as pro-inflammatory cytokines such as TNF-α and IL-6 decrease the APN mRNA expression [4]. Furthermore, lower serum APN levels are observed, e.g., in T2DM (even at its onset) and in obesity [7,8]. Decreased APN levels and M1 macrophages provoke lower expression of APN receptors and APN resistance [9]. The therapeutic potential of APN is a scientific area of great interest. Study on the effect of treatment with APN revealed its i) anti-inflammatory, ii) anti-atherogenic, and iii) insulin sensitizing influence in both human and murine models, similarly to the action of thiazolidinediones [8].

Epigenetic mechanisms may transmit the effects of various environmental factors to cells by modulating gene expression. Modifications of amino acids on histone tails, such as methylation and acetylation, determine the state of chromatin condensation, thus modulating the availability of gene promoters for transcription factors. Histone marks H3K4me3 and H3K9/K14ac, located in the promoter region, result in gene transactivation, whereas enrichment of H3K9me3 represses gene transcription [10]. Another epigenetic mechanism, miRNA interference, involves short non-coding RNAs of 21-23 nucleotides in length (miRNAs). The latter are capable of regulating mRNA levels and their translation rate either by complementary or almost complementary binding to the mRNA sequence and usually suppress its translation. There are studies showing that the expression of miRNAs is sensitive to HG, and their deregulation contributes to IR, inflammation, and oxidative stress [11]. Some miRNAs, such as plasma miR-193b-3p, are even proposed as markers of prediabetes [12]. Thus, investigation of adipokine dysregulation at the epigenome level could be a promising research field regarding the pathogenesis and potential therapeutic targets for impaired glucose metabolism (IGM) and MetS. Thus, it is tempting to speculate whether preadipocytes (pAds) present in VAT may be susceptible to a harmful hyperglycemic (HG) environment and whether subsequent generations of Ads may manifest aggravated metabolic deregulation.

The aim of this research was to investigate the expression patterns of IL-6 and APN during the adipogenesis of the HPA-v cell line (human visceral preadipocytes) and to explore the alterations induced by chronic and transient HG exposure at each adipogenesis stage. Moreover, our research aims to assess the concomitant epigenetic changes: histone modifications within promoter regions of examined genes (H3K4me3, H3K9/K14ac, H3K9me3) and the expression patterns of miRNAs previously proven or predicted to modulate the expression of the adipokines either directly or indirectly.

## 2. Results

**Figure 1:**
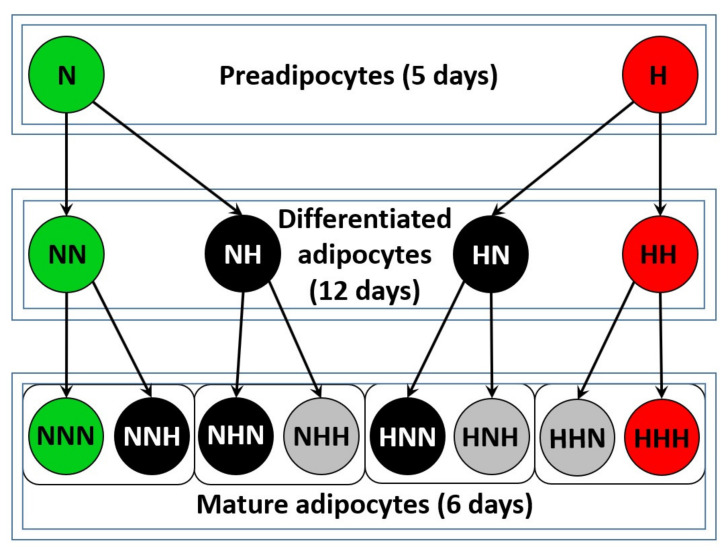
Diagram presenting the human visceral preadipocyte (HPA-v) cell culture research model. Variants of adipogenesis in chronic normoglycemia or hyperglycemia (HG) are colored green and red, respectively. Cells treated with transient HG are colored according to the number of HG stimuli: black denotes single HG stimulus, grey denotes double HG stimuli.

Adipokine expression and epigenetic modifications were assessed in HPA-v cells differentiating in normoglycemia (NG) or exposed to chronic and transient HG. The research design is presented in Figure 1. 

Each variant was coded with the letters N (for NG) or H (for HG), reflecting the glycemic conditions, whereas the number of letters denoted the number of completed stages of cell culture (e.g., NNN refers to mature adipocytes that were cultured in NG for all three stages of adipogenesis; HN refers to cells after the differentiation stage that were exposed to HG during the first stage—preadipocyte proliferation).

### 2.1. Expression Profiles of IL-6 and APN Are Disrupted with Chronic HG Exposure

**Figure 2:**
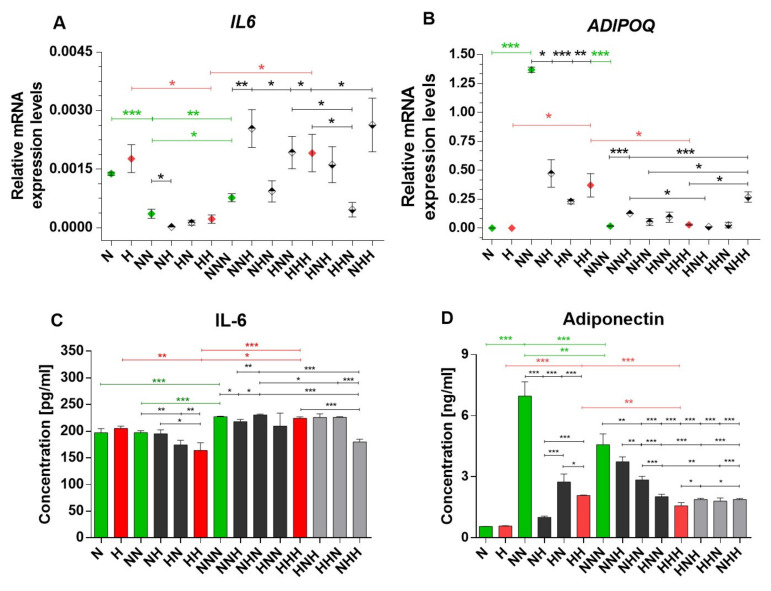
Expression of *IL6* (**A**), *ADIPOQ* (**B**), mRNAs and IL-6 (**C**), and APN (**D**) proteins in studied human visceral preadipocytes (HPA-vs). Variants differentiated chronically in normoglycemic conditions and chronic hyperglycemia (HG) are colored in green and red (rhombuses or bars), respectively. In Figure 2A,B, variants of transient HG treatment applied during one stage of cell culture are marked by rhombuses with a black upper half and variants exposed to HG during two stages are denoted as rhombuses with a black lower half. In Figure 2C,D, variants of transient HG treatment applied during one stage of cell culture are denoted by black bars and variants exposed to HG during two stages are denoted by grey bars. Data are presented as mean ± SEM. Significant differences are marked by asterisks according to the *p*-value criteria: *—0.05 ≥ *p* > 0.01, **—0.01 ≥ *p* > 0.001, ***—*p* ≤ 0.001. Significant differences estimated in one-way ANOVA with a post-hoc Tukey test are marked by green or red asterisks.

We observed that, during adipogenesis under NG, the expression of IL-6 mRNA significantly decreased after adipocyte differentiation (NN vs. N, *p* < 0.001) and was slightly elevated after adipocyte maturation (NNN vs. NN, *p* = 0.0068; Figure 2A). However, the IL-6 mRNA level in mature Ads was lower than in pAds (NNN vs. N, *p* = 0.0458). Chronically HG-treated Ads manifested similar IL-6 mRNA expression patterns (HH vs. H, *p* = 0.0471; HHH vs. HH, *p* = 0.0332). Overall, HG-treated mature Ads had a higher IL-6 mRNA level than untreated Ads (HHH vs. NNN, *p* = 0.0416), whereas in preadipocytes, the transcription of IL-6 only tended to increase (H vs. N).

The level of IL-6 protein in mature Ads cultured chronically in NG was elevated as compared to previous culture stages (*p* < 0.001 for both NNN vs. NN and NNN vs. N; Figure 2C). IL-6 protein levels in Ads treated with chronic HG reflected the mRNA expression (HH vs. H, *p* = 0.0013; HHH vs. HH, *p* < 0.001), although IL-6 was produced more abundantly in mature Ads (HHH vs. H, *p* = 0.0493). Accordingly, after the differentiation stage, the IL-6 protein level was downregulated in hyperglycemic adipogenesis in comparison to NG adipogenesis (HH vs. NN, *p* = 0.0062).

After the differentiation stage, transient HG treatment caused the downregulation of the IL-6 mRNA expression in the NH variant (NH vs. NN *p* = 0.0482), whereas the protein level was decreased in the HN variant (HN vs. NN, *p* = 0.0051), imitating the effects of chronic HG. In mature Ads, a single stage of HG treatment during either the preadipocyte proliferation or maturation stage resulted in increased IL-6 mRNA levels (HNN vs. NNN, *p* = 0.0249; NNH vs. NNN, *p* = 0.0061, respectively). Double-stage HG treatment of Ads (during the differentiation and maturation stages) induced more abundant IL-6 mRNA expression (NHH vs. NNN, *p* = 0.0141). Interestingly, double-stage exposure to HG in the HHN variant resulted in a lower IL-6 mRNA level than in chronically HG-treated Ads (HHN vs. HHH, *p* = 0.0483), whereas the expression level was also lower than after HG treatment only during the proliferation stage (HHN vs. HNN, *p* = 0.0319).

Next, the IL-6 protein level in mature Ads treated with HG during a single stage of adipogenesis was downregulated when HG occurred solely in the maturation stage (NNH vs. NNN, *p* = 0.0207), yet it was upregulated when HG was implemented in the differentiation stage (NHN vs. NNN, *p* = 0.0381). Interestingly, Ads exposed to HG during the differentiation and maturation stages had lower IL-6 protein levels than both HG-treated variants in these stages separately (NHN vs. NHH, *p* < 0.001 and NNH vs. NHH, *p* < 0.001). Furthermore, in the mentioned variant, the IL-6 level was reduced as compared to chronically HG-treated Ads (HHH vs. NHH, p < 0.001) and to the NG variant (NHH vs. NNN, *p* < 0.001). 

In summary, either chronic or transient HG enhanced the transcription of IL-6 in mature Ads, whereas, at protein level, both chronic and transient HG induced a notable decrease after the differentiation stage.

In NG conditions, we observed that the mRNA expression of APN was highly increased after adipocyte differentiation (NN vs. N; *p* < 0.001) and diminished pronouncedly in mature Ads (NNN vs. NN; *p* < 0.001; Figure 2B). A similar expression pattern occurred in chronically HG-treated HPA-v cells (HH vs. H, *p* = 0.0212; HHH vs. HH, *p* = 0.0435), instead of a notably lower differentiation stage peak (HH vs. NN, *p* = 0.0047). Although not clearly shown in Figure 2 and indicated by ANOVA, mRNA expression of APN slightly increased in mature Ads both in NG and HG (NNN vs. N, HHH vs. H; t-test *p* = 0.0153, *p* = 0.0037, respectively). Moreover, APN protein levels were elevated after the induction of adipogenesis in subsequent stages of cell culture in both NG and HG conditions (NN vs. N, NNN vs. N, HH vs. H, HHH vs. H, *p* values < 0.001). Chronically HG-treated Ads had also lower APN levels after the differentiation (HH vs. NN, *p* < 0.001) and maturation (HHH vs. NNN, *p* < 0.001) stages than in control variants (Figure 2D).

Both mRNA (NH vs. NN, *p* = 0.0102; HN vs. NN, *p* < 0.001) and protein (NH vs. NN, HN vs. NN, *p* < 0.001) APN expression in both transiently HG-treated variants after adipocyte differentiation were lower than in the control, resembling the effects of chronic HG. Interestingly, in mature Ads exposed to a single stage of HG treatment, APN protein expression was lower than in the control (NHN vs. NNN, HNN vs. NNN; *p* = 0.0055, *p* < 0.001, respectively), apart from the NNH variant. However, in every variant of Ads subjected to HG treatment during two stages of adipogenesis, APN decreased to the level of chronically HG-treated Ads (HNH vs. NNN, HHN vs. NNN, NHH vs. NNN; *p* < 0.001 for all). For double-stage treatment with HG, APN protein levels were also lower than in cells treated with transient HG during a single stage of the cell culture (e.g., NHH vs. NNH, NHH vs. NHN, HNH vs. NNH; *p* < 0.001 for all). To conclude, mRNA and protein APN expression in NG were significantly upregulated after the differentiation stage and then diminished in mature Ads. In chronic and transient HG, the mRNA and protein expression pattern of APN was similar to chronic NG, but at a notably lower level.

### 2.2. Analysis of Alterations in Selected Post-Translational Histone Modifications in the IL6 and ADIPOQ Promoter Regions

**Figure 3:**
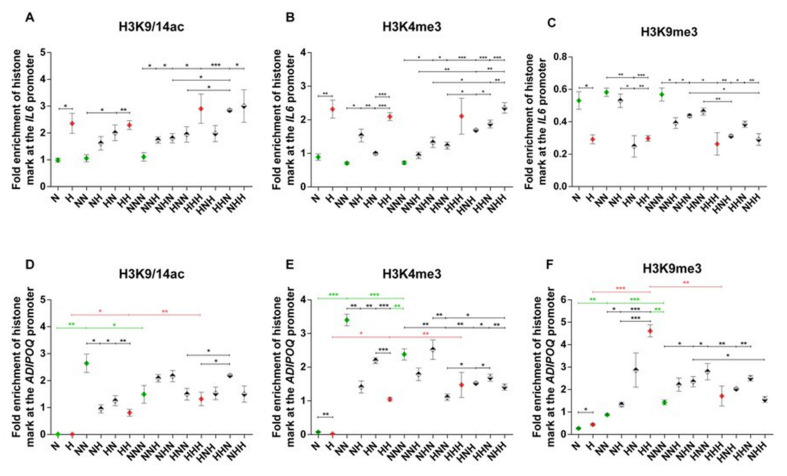
Fold enrichment of H3K9/K14ac, H3K4me3, and H3K9me3 marks in region 1 in the gene promoters of *IL6* (**A**–**C**) and *ADIPOQ* (**D**–**F**) in studied human visceral preadipocytes (HPA-vs). Variants differentiated chronically in normoglycemic conditions and chronic hyperglycemia (HG) are colored in green and red, respectively. Variants of transient HG treatment applied during one stage of cell culture are marked with rhombuses with a black upper half and variants exposed to HG during two stages are denoted by rhombuses with a black lower half. Data are presented as mean ± SEM. Significant differences are marked by asterisks according to the *p*-value criteria: *—0.05 ≥ *p* > 0.01, **—0.01 ≥ *p* > 0.001, ***—*p* ≤ 0.001. Significant differences estimated in one-way ANOVA with a post-hoc Tukey test are marked by green or red asterisks.

The analyzed histone modifications at the *IL6* gene promoter were at stable levels during adipogenesis in both NG and HG (Figure 3A–C). Furthermore, throughout adipogenesis in chronic HG, active chromatin marks were persistently enhanced following the proliferation stage, albeit only with a trend for increased H3K4me3 while comparing HHH and NNN variants. Consequently, the enrichment of H3K9me3 decreased upon chronic HG at each culture stage (H vs. N, HH vs. NN, HHH vs. NNN), highlighting the convergent direction of changes in active and repressive chromatin marks within the *IL6* promoter region. After the differentiation stage, most of the transiently HG-treated HPA-v variants manifested histone marks that were altered consistently with the influence of chronic HG, e.g., H3K9/14ac and H3K9me3 levels, in HN variants when compared to NN cells. We observed that changes in histone marks may mostly underlie the mRNA expression changes in IL-6 in HG-treated pAds and mature Ads, while data regarding changes detected at the second stage of cell culture may also imply the impact of other types of epigenetic modifications. 

Moreover, we detected a comparable increase in active chromatin marks and decrease in H3K9 trimethylation in most variants of the mature Ads exposed to transient HG (e.g., NHH vs. NNN), with often an even stronger effect of double-stage HG treatment (e.g., NHH vs. NHN). The histone modifications described above were in line with dysregulated levels of IL-6 mRNA in many cases of transiently HG-treated mature Ads. Moreover, histone modifications covered by chromatin immunoprecipitation (ChIP) in two other promoter regions were in agreement with either the main findings considering region 1 or altered IL-6 mRNA expression (Supplementary Figure S1).

During adipogenesis in NG, we detected upregulated enrichment of all histone modifications at the *ADIPOQ* gene promoter after the differentiation stage (NN vs. N; Figure 3D-F). The following stage resulted in a decline in H3K4me3 (with a trending decline in acetylation) and elevated H3K9me3 (NNN vs. NN). However, in mature Ads, all histone modifications were induced as compared to pAds (NNN vs. N). In HG, active chromatin marks were similarly enhanced, with a substantial elevation of H3K9me3 after the differentiation stage (HH vs. H). Upon maturation, only H3K9me3 was reduced (HHH vs. HH). Overall, the levels of examined active histone modifications were consistent with APN mRNA expression during adipogenesis in NG, including those presented in Supplementary Figure S2.

Considering the impact of HG on pAds, we observed slightly decreased H3K4me3 and increased H3K9me3 along with an absence of changes in H3K9/14 acetylation (H vs. N). In differentiated Ads, the levels of active chromatin marks was reduced and repressive H3K9me3 was markedly enhanced by HG as compared to NG, which reflected the declined level of APN mRNA (HH vs. NN). Transient HG treatment in this stage induced comparable changes in histone marks (NH vs. NN, HN vs. NN), which strongly corresponded with APN mRNA levels. Moreover, ChIP analysis of other examined promoter regions (Supplementary Figure S2) indicated that the histone marks’ patterns induced upon chronic and transient HG after the second stage of HPA-v culture were convergent with the changes detected in region 1.

No significant differences in the levels of histone modifications triggered by chronic HG were found in mature Ads (HHH vs. NNN). In the case of transient HG, we observed no significant influence on acetylation of H3K9/14 in comparison to NG (NNN). However, chronic (HHH) and single-stage HG treatment (HNN) showed downregulation of H3K9/14 acetylation in comparison to the HHN variant. The level of another active chromatin mark, H3K4me3, was decreased or showed a tendency to decrease in most cases of HG-exposed variants, with the exception of NHN. We also detected several significant differences in the levels of H3K4me3 while comparing variants with a double vs. single HG hit (e.g., NHH vs. NHN). Inversely, in the majority of transient HG variants, inhibitory H3K9me3 was upregulated or manifested a trend for an increase. The only exception was the NHH variant, which demonstrated a reduction in the trimethylation of H3K9 when compared to the NHN variant, being in line with the regulation of the APN mRNA level. Taking all marks into account, HG could induce rather suppressive histone modifications at the third stage of adipogenesis, which was not entirely consistent with the APN mRNA expression pattern.

### 2.3. miRNA Expression Analysis

**Figure 4:**
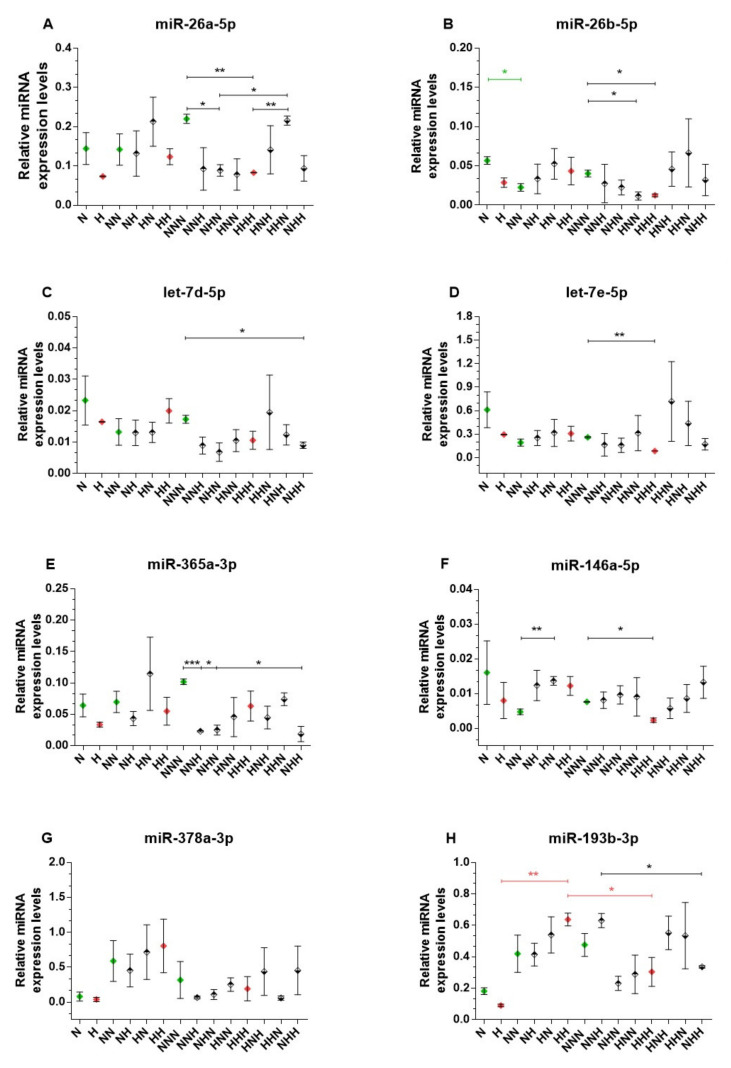
Expression of selected miRNAs in studied cell culture model (**A**–**H**). Variants differentiated in normoglycemic conditions and chronic hyperglycemia (HG) are colored in green and red, respectively.Variants of transient HG treatment applied during one stage of cell culture are marked with rhombuses with a black upper half and variants exposed to HG during two stages are denoted by rhombuses with a black lower half. Data are presented as mean ± SEM. Significant differences are marked by asterisks according to the *p*-value criteria: *—0.05 ≥ *p* > 0.01, **—0.01 ≥ *p* > 0.001, ***—*p* ≤ 0.001. Significant differences estimated in one-way ANOVA with a post-hoc Tukey test are marked by green or red asterisks.

After comparative analysis of the histone marks and mRNA expression patterns of the examined genes, we decided to investigate another epigenetic mechanism. Thus, we selected miRNAs validated or predicted to target IL-6 and APN mRNAs, as described in the Methods section. Aside from miRNAs directly targeting the studied genes, we investigated also related miRNAs known to indirectly regulate IL-6 and APN. MiR-146a-5p affects the NFkB-IL-6 axis through its direct targets, IRAK1 and TRAF6 [13]. Additionally, we measured the expression of miR-193b-3p, which was found to indirectly upregulate APN expression (through NFYA and NRIP1 transcription factors) [14]. The expression of the studied miRNAs is presented in Figure 4. Among the miRNAs related to IL-6, only the level of miR-26b-5p changed during adipogenesis in NG conditions (downregulation NN vs. N, *p* = 0.0165; Figure 4B). Moreover, we detected that mature Ads exposed to HG during the differentiation stage had decreased expression of miR-26a-5p, miR-26b-5p, miR-146a-5p, and let-7e-5p in comparison to untreated Ads (HHH vs. NNN; *p*-values: *p* = 0.0073, *p* = 0.0274, *p* = 0.0144, *p* = 0.0022, respectively). Such HG-induced downregulation was in line with the transactivation of IL-6.

After the differentiation stage, only miR-146a-5p levels were elevated in HG-treated Ads during the preadipocyte proliferation stage (HN vs. NN, *p* = 0.004; Figure 4F). Later on, in mature Ads, a single stage of transient HG treatment resulted in reduced levels of miR-26a-5p (NHN vs. NNN, *p* = 0.0199), miR-26b-5p (HNN vs. NNN, *p* = 0.0311), and miR-365a-3p (NNH vs. NNN, *p* < 0.001; NHN vs. NNN, *p* = 0.0133).

Additionally, in Ads exposed to HG during the differentiation and maturation stages, the expression of miR-365-3p and let-7d-5p was lower in comparison to the control (NHH vs. NNN, *p* = 0.0233 and *p* = 0.0363, respectively). However, in HG-treated Ads during first two stages of the cell culture, the level of miR-26a-5p was not different from the control. Moreover, in the mentioned variant, the miR-26a-5p level was higher than in the case of chronic HG exposure (HHH vs. HHN, *p* = 0.0075) or after HG treatment only during the differentiation stage (HHN vs. NHN, *p* = 0.021).

The expression of miR-378a-3p (predicted as potentially targeting APN mRNA) was unchanged among all studied variants (Figure 4G). Moreover, the expression of miR-193b-3p was unchanged throughout the adipogenesis in NG conditions, whereas chronic HG resulted in an elevation and subsequent decrease in the miR-193b-3p level after differentiation and maturation, respectively (Figure 4H). Furthermore, we observed a decline in the miR-193b-3p level measured in double-stage HG-treated Ads (NHH) in comparison to single-stage HG-treated cells (NNH).

## 3. Discussion

Our primary aim was to determine whether exposure to chronic or transient HG could induce changes in the expression of IL-6 and APN in the studied model. We observed differences in the expression patterns between NG and chronic HG conditions during adipogenesis for both adipokines. Either chronic or transient HG affected IL-6 mRNA expression while inducing transactivation in mature Ads and triggering declined protein production after the differentiation stage. For both types of HG treatment, Ads manifested a remarkable decrease in APN expression at mRNA and protein levels after the differentiation stage and lower protein levels after the maturation stage. Significantly, after double-stage HG treatment, APN protein levels resembled the effects of chronic HG. The next step in our study was to explore changes in the epigenomes of IL-6 and APN in order to establish possible gene expression modulation induced by HG. Firstly, we performed the *IL6* and *ADIPOQ* promoter walk ChIP, which showed alterations in the levels of H3K4me3, H3K9/K14ac, and H3K9me3 upon chronic and transient HG for both studied genes at each culture stage. The observed alterations may explain some of the mRNA expression changes in IL-6 and APN. Multiple miRNAs (miR-26a-5p, miR-26b-5p, let-7e-5p, miR-146a-5p) were downregulated after adipogenesis in chronic HG, reflecting the changes in IL-6 mRNA expression. Furthermore, several miRNAs were similarly affected in transiently HG-treated HPA-vs. We also detected similar adipogenesis expression patterns of APN mRNA and miR-193b-3p in chronically HG-treated cells.

The expression patterns of IL-6 mRNA during adipogenesis in chronic HG and NG were comparable, especially due to a decrease after the differentiation stage, which, in chronic HG, was apparent also in the protein levels. This is in agreement with in vitro research showing that IL-6 may inhibit the expression of C/EBPα (the transcription factor crucial for Ads differentiation) and other adipogenic markers potentially disturbing the phenotype of Ads [15,16,17]. Therefore, the inhibition of IL-6 transcription could be protective for C/EBPα expression and Ads commitment. Consistently, transcription of *CEBPA* was opposed to the IL-6 mRNA levels in this model, whereas chronic HG accelerated adipogenesis, as we reported previously [18]. On the other hand, Chinese researchers described a gradual increase in IL-6 production during adipogenic differentiation in human bone marrow mesenchymal stem cells (BM-MSCs), while we observed increased IL-6 production in mature Ads [19]. Consistent with this, HG transactivated IL-6 in vitro and in the T2DM murine model, where mesenchymal stem cell (MSC) infusion treatment reversed these changes [20,21]. Moreover, the levels of serum IL-6 reflected the changes in its transcription [21].

In line with our results, the expression of the IL-6 protein in epicardial AT (EAT) was recently reported as unchanged in T2DM and obesity [22]. However, the authors also revealed that SAT produced IL-6 more abundantly, despite markedly lower mRNA expression than in EAT [22]. Nevertheless, plasma IL-6 showed no differences among the groups under study [22]. Unlike the EAT, enhanced IL-6 production in SAT, without changes in plasma IL-6 levels, could mean higher levels of ready-to-release IL-6 in SAT. Furthermore, EAT could represent visceral adipose tissue that has not been infiltrated by macrophages as much as typical VAT and, therefore, similar to our HPA-v model. Moreover, a recent intra-abdominal SAT implantation experiment showed that implantation of SAT could alleviate impaired glucose tolerance and lower serum IL-6 levels in mice fed a high-fat diet (HFD) [23]. On the other hand, VAT implantation had the opposite effect [23]. In contrast to our research, the epididymal AT of mice fed a high-refined-carbohydrate diet produced increased IL-6 levels [24]. Furthermore, Czech researchers showed increased serum IL-6 levels in T2DM patients, which declined after a very-low-calorie diet intervention [25]. The aforementioned reports also support the greater role of VAT in the dysregulation of pro-inflammatory cytokines in T2DM. However, the release of IL-6 could be different in our model given that macrophages are the main producers of IL-6 and abundantly infiltrate the VAT in obesity [2,26]. Indeed, most studies in cell culture models measure secreted IL-6. In the research of Youssef-Elabd et al., neither chronic nor transient HG treatment of subcutaneous Ads altered the secretion of IL-6 [27]. By contrast, Cai et al. showed elevated release of IL-6 from the 3T3-L1 Ads exposed to HG [28]. Our results did not fully support the notion that HG enhances IL-6 production in Ads. Nevertheless, visceral Ads culture models are rarely used in research on IL-6 expression and may differ from other types of Ads.

In line with our results, a few studies on human cell models confirmed decreased APN expression after exposure to HG during the differentiation stage [29,30,31].

In contrast, Jackson et al. reported that a 25 mM pulse of glucose was required at the third day of 3T3-L1 Ads differentiation, which expressed APN in a glucose-dependent manner. Therefore, the authors suggested that HG contributes to AT hyperplasia [32]. This different approach merits attention, but the results are difficult to compare. Firstly, 3T3-L1 is a murine embryonic cell line, whereas HPA-v cells were obtained from the AT of Caucasian adults and varied in differentiation length. Assuming diverse stages of commitment of cells and distinct differentiation inducers, more evidence regarding adult-derived cell lines is needed [33]. Apart from model differences, scientists used 4 mM of glucose as NG. However, 3T3-L1 cells needed at least 9 mM of glucose to produce APN, which is a significant difference in culture conditions. The most important stage of differentiation requiring the glucose pulse was also assessed using a design reflecting our transient HG exposure, but with contrasting results [32]. Indeed, the authors observed around 75% lower lipid accumulation in 4 mM of glucose, and, previously, we had consistently observed that HG accelerated adipogenesis [18]. Thus, more research is needed to explore the concentration of glucose dysregulating adipokines and Ads metabolism. Furthermore, Shilpa et al. reported a strikingly lower APN protein level in 3T3-L1 Ads differentiated in 105 mM of glucose [34]. Research on APN expression involving human cell lines is much less common than that on the 3T3-L1 cell line. Moreover, the aforementioned reports varied in the duration of adipogenesis and the applied glucose concentrations. Chang et al. observed a decreased APN mRNA level after one month of HG treatment before differentiation. Although we detected no such change, these results are in accordance with the declined protein levels in mature Ads treated with HG only during proliferation [35].

Studies on mature Ads consistently demonstrated reduced APN protein expression after exposure to HG for either 2 or 7 days [36,37]. Surprisingly, Chinese researchers demonstrated that constant HG impact for 72 h limited APN expression, while HG fluctuating every 6 hours evoked even stronger restraint [38]. We also observed decreased APN expression after transient HG treatment, especially at the protein level. The results regarding mature Ads exposed to HG for a certain period of time more clearly show impaired adipokine expression, yet most of the reports examining the effects of HG exposure during differentiation did not measure the long-term effects of HG in mature Ads. Future research concerning HG-deregulated adipokine expression in Ads could evaluate the impact of HG on the secretion and intracellular levels of adipokines in differentiating human visceral Ads, possibly in comparison to subcutaneous Ads. Moreover, further research could investigate the long-term effects of HG on APN expression (after adipogenesis).

HG introduced persistent changes in H3K4me3, H3K9/K14ac, and H3K9me3 marks starting from pAds to mature Ads in the promoter of the *IL6* gene. Moreover, either chronic or transient HG treatment of mature Ads exhibited similar enrichment of histone marks concomitantly with abundant *IL6* transactivation in most variants of mature Ads. These results may imply the metabolic memory of the impact of HG in visceral Ads. However, changes in the levels of histone marks were not reflected in IL-6 mRNA expression at the stage of differentiated Ads. This may suggest that some other epigenetic mechanisms (e.g., miRNAs, lncRNAs, DNA, and RNA methylation) may also govern the IL-6 mRNA expression at this stage of adipogenesis.

To the best of our knowledge, changes in histone marks within the *IL6* promoter have not been investigated during adipogenesis yet. Recently, the H3K4me3 mark at the *IL6* promoter and mRNA transcription were found to be upregulated in the VAT of morbidly obese prediabetic subjects in comparison to normoglycemic subjects, which is in line with our data [39].

Among the studied histone modifications within the *ADIPOQ* gene promoter, the levels of H3K4me3 and H3K9/K14ac were convergent with APN mRNA expression patterns during adipogenesis in NG. Thus, these histone marks may serve as the dominant epigenetic modulatory mechanism in controlling the mRNA expression of APN, a well-known adipogenesis marker, during the process of physiological Ads development. Analyzing adipogenesis in HG, we observed that changes in active and repressive histone marks were not convergent with each other; therefore, it was difficult to discern a clear correlation with the APN mRNA pattern.

We also detected some significant changes in histone methylation marks in pAds upon HG, yet they were probably too subtle to evoke changes in APN transcription. By contrast, the dramatic downregulation of APN mRNA expression in all HG-treated variants of differentiated Ads was accompanied by downregulation of active chromatin marks and upregulation of H3K9me3 levels. Considering mature Ads, HG seemed to induce, rather suppressive methylation marks, while not affecting H3K9/14ac. Therefore, the mostly stable APN mRNA expression could be a result of the interplay with other epigenetic modifications, including those mentioned above, as well as unexamined histone marks (e.g., H3K27me3, H4K14ac).

Simultaneous peaks in H3K9/14ac and H3K4me3 marks and APN transcription during adipogenesis of 3T3-L1 Ads, along with the positive correlation between these parameters, described by Musri et al., constitute strong evidence supporting our results [40]. Furthermore, research on the murine model reported that the obesogenic and diabetogenic influence of in utero HFD exposure induced concomitant imprinted deacetylation of H3K9 at the *ADIPOQ* gene promoter [41].

Aside from the evaluation of histone modifications, we also measured the expression of several miRNAs that could either directly or indirectly influence the mRNA and protein expression of IL-6 and APN. Firstly, the differences between the IL-6 protein and mRNA expression in NG adipogenesis may arise from post-transcriptional modifications, e.g., during preadipocyte proliferation. Indeed, we observed that the level of miR-26b-5p was higher after the first stage as compared to the differentiation stage (NN vs. N). In turn, Ortega et al. detected upregulation of miR-365, let-7d-5p, and miR-378a during the adipogenesis of human subcutaneous Ads, in contrast to the unchanged expression observed in our HPA-v model [42]. However, in chronic HG, the protein levels in mature Ads were unaffected, unlike the miRNA levels. Thus, in chronic HG conditions, translation of IL-6 could be diminished by some other mechanism. 

A number of studies estimating plasma miRNAs in diabetic patients reported reduced levels of some of our studied miRNAs (miR-26a-5p, miR-365a-3p) [43,44]. On the other hand, we previously reported upregulated let-7d-5p, let-7e-5p, and miR-365a-3p in the VAT of female T2DM and impaired fasting glucose participants. In addition, diabetic liver and skeletal muscles had elevated let-7d-5p and let-7e-5p levels [45,46]. However, in prediabetic patients, dietary and exercise interventions reduced circulatory let-7e-5p [47]. Recently, Cirilli et al. observed that high levels of physical activity alleviated blood pressure, HbA1c, and VAT/total AT ratio and decreased plasma miR-146a-5p levels in T2DM patients [48].

Cellular and tissular miRNA levels may differ, as AT contains, e.g., macrophages interacting with other cells [2]. It is noteworthy that, in the aforementioned research on IGM, the dysregulated circulatory miRNAs levels support our results, whereas most of the reports that do not support our findings describe tissular miRNAs levels.

According to our results, overexpression of miR-26 in mice protected against HFD-induced obesity, while miR-26 deficiency resulted in AT hyperplasia [49]. More recently, Shi et al. observed that elevated miR-26a-5p mediates the protective effect of Ginsenoside Rg1 against HG toxicity in human ARPE-19 (retinal pigment epithelial) [50]. In 2014, Chinese scientists consistently observed an HG-induced decrease in miR-26b-5p levels in HPA-v cells [51]. Furthermore, adipokines related to inflammation and IR (excluding IL-6) also reduced the expression of this miRNA in Ads [52]. Zhang et al. found that Chinese medicine used in T2DM treatment elevated serum let-7e-5p in diabetic rats [53]. Conversely, HG and high insulin transactivated let-7e-5p and downregulated IRS2 in HepG2 cells [45]. In 2014, Wang et al. revealed that HG combined with thrombin i) reduced miR-146a-5p expression, ii) induced ROS generation, and iii) transactivated *NOX4* and *IL6* in aortic endothelial cells [54]. Furthermore, transfection with miR-146a-5p protected retinal endothelial cells (REC) from DNA fragmentation under HG conditions by exerting an impact on IL-6 signaling and reduced cellular IL-6 [55]. Additionally, Roos et al. reported transactivation of miR-146a-5p in the SAT of obese subjects; however, miR-146a-5p transfection prevented the inflammatory response and decreased IL-6 mRNA expression in SGBS Ads [56]. According to Chinese scientists, miR-146a-5p could promote adipogenic differentiation in BM-MSCs, which supports the notion that IL-6 suppression is potentially required in adipogenesis [57]. 

In many cases, research results on miRNAs targeting IL-6 mRNA were in line with our findings in terms of, e.g., HG, inflammation, T2DM, and obesity, while studies that were not in line with our results focused on HFD or HepG2 cells. Indeed, many reports studied the expression of selected miRNAs in IGM, obesity, and inflammation. However, only a small number of publications analyzed IL-6 in a similar research model. Additionally, the aforementioned studies highlighted the impact of miRNAs on oxidative stress. Previously, we also reviewed the role of miR-26a-5p and miR-146a-5p in MetS-related oxidative stress [11].

In contrast to our results, Gerin et al. found miR-378 to be highly induced during the adipogenesis of 3T3-L1 cells [58]. Indeed, Yu et al. showed that miR-378 promoted the adipogenesis of human subcutaneous Ads, yet such an effect was not observed in the visceral Ads [59]. In our model, no significant expression change in miR-378a-3p was found. However, we demonstrated similar expression patterns of APN mRNA and miR-193b during adipogenesis in chronic HG conditions. This was in agreement with the indirect upregulation found by Belarbi et al.; however, our results suggested such an effect only under HG. Other reports supported a protective role of miR-193b-3p against inflammation, consistent with potentially upregulated APN [60,61].

## 4. Conclusions

To summarize, our study indicated that mRNA and protein expression of IL-6 and APN changes during NG and HG affected visceral adipogenesis. Moreover, chronic HG interfered with either mRNA or protein expression patterns of IL-6 and APN in differentiated and/or mature Ads, with a dramatic reduction in APN production in mature Ads. Transient HG upregulated IL-6 mRNA levels and downregulated APN protein levels in Ads.

Histone modifications on the promoter of *IL6* gene induced in HPA-vs by chronic and transient HG were convergent with altered transcription in most of the studied variants. Active chromatin modifications concerning the *APN* gene promoter were strongly in line with the mRNA expression patterns observed during adipogenesis in NG, while changes in all the studied histone marks supported HG-triggered APN mRNA changes in all variants of differentiated adipocytes. 

HG-induced changes in miRNA expression reflected the IL-6 mRNA expression pattern, thus supporting their putative potential to target IL-6. Additionally, the data obtained suggested that indirectly regulating miRNAs could also contribute to changes in the expression of IL-6 and APN genes. 

Altogether, HG may take advantage of different epigenetic mechanisms to affect the expression of adipocytokines in visceral Ads. Thus, the described dysregulation of epigenetic mechanisms could be instrumental in the pathogenesis of a low-grade chronic inflammation state and concomitant metabolic deregulation in VAT upon HG.

## 5. Materials and Methods

### 5.1. Cell Culture

Human visceral pAds (HPA-v) cells were cultured according to previously described procedures [62]. Cells and reagents for cell cultures were obtained from ScienCell Research Laboratories (Carlsbad, CA, USA). PAds were differentiated to mature Ads in three stages, proliferation (5 days), differentiation (12 days), and maturation (6 days), with culture in preadipocyte medium (PAM), preadipocyte differentiation medium (PADM), and adipocyte medium (AdM) media in subsequent stages, respectively. Completion of every stage of cell culture was confirmed with BODIPY 505/515 staining (Life Technologies, Eugene, OR, USA) as described previously [18]. To create hyperglycemic conditions, full medium was supplemented with glucose to obtain a concentration of 30 mM (d-(+)-Glucose, Sigma-Aldrich, Saint Louis, MO, USA). During adipogenesis, HPA-v cells were exposed to hyperglycemic conditions in a chronic and transient manner during particular culture stages, which resulted in fourteen variants at different developmental stages (Figure 4). Each variant was denoted by the letters N (5.5 mM of glucose) or H (30 mM of glucose), reflecting the glycemic conditions during subsequent completed stages of cell culture. Hence, we obtained study variants of chronic (e.g., H, HH, HHH) and transient exposure to HG (e.g., NNH–HPA-v cells cultured in 5.5 mM glucose during preadipocyte proliferation and differentiation, whereas, during adipocyte maturation, the cells were cultured in 30 mM glucose). The latter were designed to investigate: (i) the impact of exposure to HG on particular stages of adipogenesis, (ii) which stage of cell culture is critical to changes in the regulation of IL-6 and APN expression, (iii) how normalization of glucose levels influences the expression of IL-6 and APN mRNA. Cells were harvested after completion of every stage and processed as described below for particular analyses. Further analyses were based on data from three independent experiments.

### 5.2. Isolation of RNA and RT-qPCR Analysis

RNA was isolated from HPA-v cells using AllPrep DNA/RNA/Protein Mini kit (QIAGEN, Hilden, Germany). Approximately 6 × 10^6^ of harvested cells were lysed by RLT buffer with B-mercaptoethanol and homogenized by several stages of pipetting with a 20-gauge (0.9 mm diameter) needle attached to an RNase-free syringe. Next, RNA isolation was carried out according to the manufacturer’s instructions. Concentration and purity of obtained RNA was measured by Synergy HT microplate reader (Biotek, Winooski, VT, USA) and RNA was stored at -80 °C for further processing. Reverse transcription of isolated RNA was conducted using High-Capacity cDNA Reverse Transcription Kit (Applied Biosystems, Foster City, CA, USA) using Tpersonal Thermocycler (Biometra, Göttingen, Germany). Expression of mRNA of studied genes was measured by Quantitative Real-Time PCR, using TaqMan Gene Expression Assay Hs00174131_m1 for *IL6* gene and Hs00605917_m1 for *ADIPOQ* and TaqMan Gene Expression MasterMix (Applied Biosystems, Foster City, CA, USA). The procedure was executed in the following steps: UNG incubation (50 °C, 2 min), polymerase activation (95 °C, 10 min), and 40 PCR cycles comprising denaturation, 95 °C, 15 s and anneal/extend, 60 °C, 1 min. Data were calculated using the 2−ΔCt method and normalized to the arithmetic average of Ct values obtained for *RLPLO* (ribosomal protein lateral stalk subunit P0) and *UBC* (ubiquitin C). The most stable genes were chosen on the basis of expression-profiling of 16 reference genes in each study variant, on the basis of several analyses performed in RefFinder [63]. Again, equalized RNA amounts were converted to cDNA using the reagents mentioned above and expression-profiling was done using TaqMan Array Human Endogenous Control Plates (Applied Biosystems, Foster City, CA, USA) in accordance with the manufacturer’s protocol (data not shown).

### 5.3. miRNA Isolation and Expression Profiling

Isolation and miRNA profiling were described previously [62]. In short, trypsinized cells were lysed in Lysis/Binding buffer and miRNA was isolated according to the protocol of miRVANA Isolation Kit (Applied Biosystems, Vilnius, Lithuania). TaqMan® MicroRNA Reverse Transcription Kit and Custom RT Primer Pool (Applied Biosystems, Foster City, CA, USA) were used in reverse transcription reaction. Expression profiling of miRNAs was carried out with TaqMan Low-Density Array (TLDA) cards (Applied Biosystems, Foster City, CA, USA) using 7900HT Fast Real-Time PCR System (Applied Biosystems, Foster City, CA, USA). miRNA expression data were determined from duplicate results, normalized using the 2−∆Ct method with the arithmetic average of Ct values for reference genes let-7b-5p (002619) and *U6* (001973) selected using RefFinder software. The presented miRNAs were chosen based on screening analysis, bioinformatics analysis as well as literature search (miR-26a-5p assay ID 000405, miR-26b-5p assay ID 000407, let-7d-5p assay ID 002283, let-7e-5p assay ID 002283, miR-365a-3p assay ID 001020, miR-146a-5p assay ID 000468, miR-378a-3p assay ID 002243, mir-193b-3p assay ID 002367; Applied Biosystems, Foster City, CA, USA).

### 5.4. Protein Isolation and Enzyme-Linked Immunosorbent Assay (ELISA)

Isolation of HPA-v cells’ total protein involved the use of RIPA buffer with addition of Pierce mini protease and phosphatase inhibitors tablets (Thermo Scientific, Rockford, IL, USA). Trypsinized and washed (thrice with DPBS) HPA-vs were incubated for 15 min on ice and then centrifuged (15,000 rpm, 4 °C, 15 min). Protein concentration in collected supernatant was measured using Protein Determination Kit (Caymann, Ann Arbor, MI, USA) and isolated proteins were stored at −80 °C for further processing. Expression of IL-6 and APN proteins was measured by ELISA method (IL-6, product no. SEA079Hu; adiponectin, product no. SEA605Hu; Cloud-Clone Corp, Katy, Texas, USA), according to the protocol.

### 5.5. Chromatin immunoprecipitation (ChIP) and qPCR analysis

Chromatin fixation and shearing: After the incubation of cells in NG/HG conditions, the cells were fixed with 1% formaldehyde (10 minutes) followed by quenching in 125 mM glycine. Cells were washed in cold PBS and harvested by cell scraper in ice cold PBS with protease inhibitors (Thermo Scientific, Rockford, IL, USA) and then resuspended in SDS Lysis Buffer (0.5% SDS, 10 mM EDTA,50 mM Tris-HCl pH 8.1, with protease inhibitors). Chromatin was sheared by sonication (Bioruptor; Diagenode, Liège, Belgium) to generate fragments of 200–600 bp (40 min of sonication using a 30 s “on and off” cycle scheme; high power). Samples were centrifuged for 10 minutes at 4 °C at 13 000 g to separate the sheared chromatin from the cell debris. The sonication efficiency was checked by DNA electrophoresis. ChIP reaction: We used Magna ChIP™ A/G Chromatin Immunoprecipitation Kit (Merck, Darmstadt, Niemcy). 1 µg of DNA (sheared chromatin) was taken to analyze the histone acetylation/methylation pattern in the presented experimental conditions (2% of chromatin was saved for qPCR reaction as an input). The chromatin samples were diluted with ChIP dilution buffer (0.01% SDS, 1.1% Triton X-100, 1.2 mM EDTA, 16.7 mM Tris-HCl pH 8.1, 167 mM NaCl, protease inhibitors cocktail) and precleared with washed beads for 2 hours at 4 °C. Simultaneously, Dynabeads® magnetic beads (ThermoFischer, Vilnius, Lithuania) were washed with low-salt buffer (0.1% SDS, 1% Triton X-100, 2 mM EDTA, 20 mM Tris-HCl pH 8.0, 150 mM NaCl) and preincubated with the appropriate antibodies: H3K4me3 (Active Motif, La Hulpe, Belgium, Cat. # 39159), H3K9me3 (Active Motif, La Hulpe, Belgium, Cat. # 39161), H3K9/K14ac (Diagenode, Seraing Belgium, Cat.# C15410005), and H3 (Abcam, Cambridge, United Kingdom, Cat. #ab46765). Antibodies that did not bind to the beads were discarded and the purified chromatin was added to the bead samples. The samples were incubated overnight at 4 °C, in slow rotation. On the next day, beads were washed sequentially with low-salt buffer (0.1% SDS, 1% Triton X-100, 2 mM EDTA, 20 mM Tris-HCl pH 8.0, 150 mM NaCl), high-salt buffer (0.1%SDS, 1% Triton X-100, 2 mM EDTA, 20 mM Tris-HCl pH 8.0, 500 mM NaCl), LiCl buffer (0.25 M LiCl, 1% Tergitol, 1% deoxycholic acid, 1 mM EDTA, 10 mM Tris-HCl pH 8.0 buffer), TE buffer (10 mM Tris- HCl pH 8,0, 1 mM EDTA pH 8.0), and TE + 0.01% SDS for 5 min in each solution. Immuno-complexes were eluted from the beads using elution buffer (20mM Tris-HCl pH 7.5, 5 mM EDTA, 50 mM NaCl, 1% SDS). To reverse the protein-DNA cross-linking and recover the DNA, proteinase K (20 mg/ml, Thermo Scientific, Rockford, IL, USA) was added to the samples and mild heating was performed (2 h at 65 °C). Next, all the samples (including input) were purified by Qiagen MinElute Reaction Cleanup Kits (Cat. No. 28206, QIAGEN, Hilden, Germany). qPCR reaction: the ChIP samples were analyzed by Quantitative Real-Time PCR using Eco Real-Time PCR System (Illumina; San Diego, California, U.S.) and Takara Bio SYBR Green Master Mix chemistry (Takara, Otsu, Japan). The PCR reaction was as follows: an initial step of 30 s at 95 °C, followed by 40 cycles of: 5 s at 95 °C, 15 s at 60 °C. PCR primers (Table 1) were designed via PrimerBlast software (NCBI, Rockville Pike, Bethesda, MD, USA). The presence of single product was confirmed by melting-curve analysis, and data were analyzed to obtain enrichments relative to input. IgG and no antibody control were included in the analysis as an internal controls for binding reaction specificity.

### 5.6. Statistical Analysis

Expression of examined genes and enrichment in three examined histone modifications in this study were evaluated to assess: (i) their patterns in NG adipogenesis, (ii) the impact of HG either in chronic or transient manner (single- or double-stage exposure), (iii) importance of particular stages in changes exerted by HG. One-way ANOVA with a post-hoc Tukey test was used in multiple pairwise comparisons (adipogenesis in HG or NG conditions). Differences between mean values in particular variants were compared with two-tailed t-tests (H vs. N, NH vs. NN, HN vs. NN, HH vs. NN, HH vs. NH, HH vs. HN, NNH vs. NNN, NHN vs. NNN, HNN vs. NNN, HHN vs. NNN, HNH vs. NNN, NHH vs. NNN, HHN vs. HNN, HHN vs. NHN, HNH vs. NNH, HNH vs. HNN, NHH vs. NHN, NHH vs. NNH, HHH vs. NNN, HHH vs. HHN, HHH vs. HNH, HHH vs. NHH). All evaluated differences (GraphPad Prism 6.0 software, La Jolla, CA, USA) with *p* ≤ 0.05 were considered statistically significant.

## Figures and Tables

**Table 1:** The list of primers used for ChIP qPCR.

Gene Name	Primer Sequence	Localisation from TSS	Product (bp)
*IL6* (Region 1)	F1: 5′ AGCATGTATTGTGGGATTAC 3′ R1: 5′ GCCCTTGTTTATTCACCTAT 3′	−2074 to −1951	126
*IL6* (Region 2)	F2: 5′ GAAGAATGGATGACCTCACT 3′ R2: 5′ CTGTTGGGCATTTACTCAAG 3′	−1409 to −1327	83
*IL6* (Region 3)	F3: 5′ AGGACTGGAGATGTCTGAGGCTCATTCT 3′ R3: 5′ GTTCCAGGGCTAAGGATTTCCTGCACTT 3′	+30 to +164	135
*ADIPOQ* (Region 1)	F1: 5′ CCAAGGTGTTGAATGTTGCCA 3′ R1: 5′ TCTCTTGTGGAACCCAGCTC 3′	−990 to −878	113
*ADIPOQ* (Region 2)	F2: 5′ TTTGCCCCATCTTCTGTTGC 3′ R: 5′ ACCCTAGGGAACCTGGTACA 3′	−281 to −154	128
*ADIPOQ* (Region 3)	F3: 5′ ATAGCCTCTGGCTGGGATCA 3′ R3: 5′ GAGGAAGAAGCCCAGTGCAT 3′	+86 to +205	120

## Data Availability

Not applicable.

## Supplementary Materials

The following are available online at https://www.mdpi.com/article/10.3390/ijms22136964/s1. Supplementary Figure S1. Fold enrichment of H3K9/K14ac (A, B; Region 2 and 3), H3K4me3 (C), H3K9me3 (D, Region 2) marks at the IL6 gene promoter (A–C) in studied human visceral preadipocytes (HPA-vs). Supplementary Figure S2. Fold enrichment of H3K9/K14ac (A, B; Region 2 and 3), H3K4me3 (C), H3K9me3 (D, Region 2) marks at the ADIPOQ gene promoter in studied human visceral preadipocytes (HPA-vs).
