# Supplementary material for: Chronic and Transient Hyperglycemia Induces Changes in the Expression Patterns of IL6 and ADIPOQ Genes and Their Associated Epigenetic Modifications in Differentiating Human Visceral Adipocytes
Source: Int J Mol Sci. 2021 Jun 28;22(13):6964. doi: 10.3390/ijms22136964 (PMC8268546; doi:10.3390/ijms22136964)
Supplement: Supplementary file 1 [file ijms-22-06964-s001.zip › ijms-1247572-supplementary.pdf]

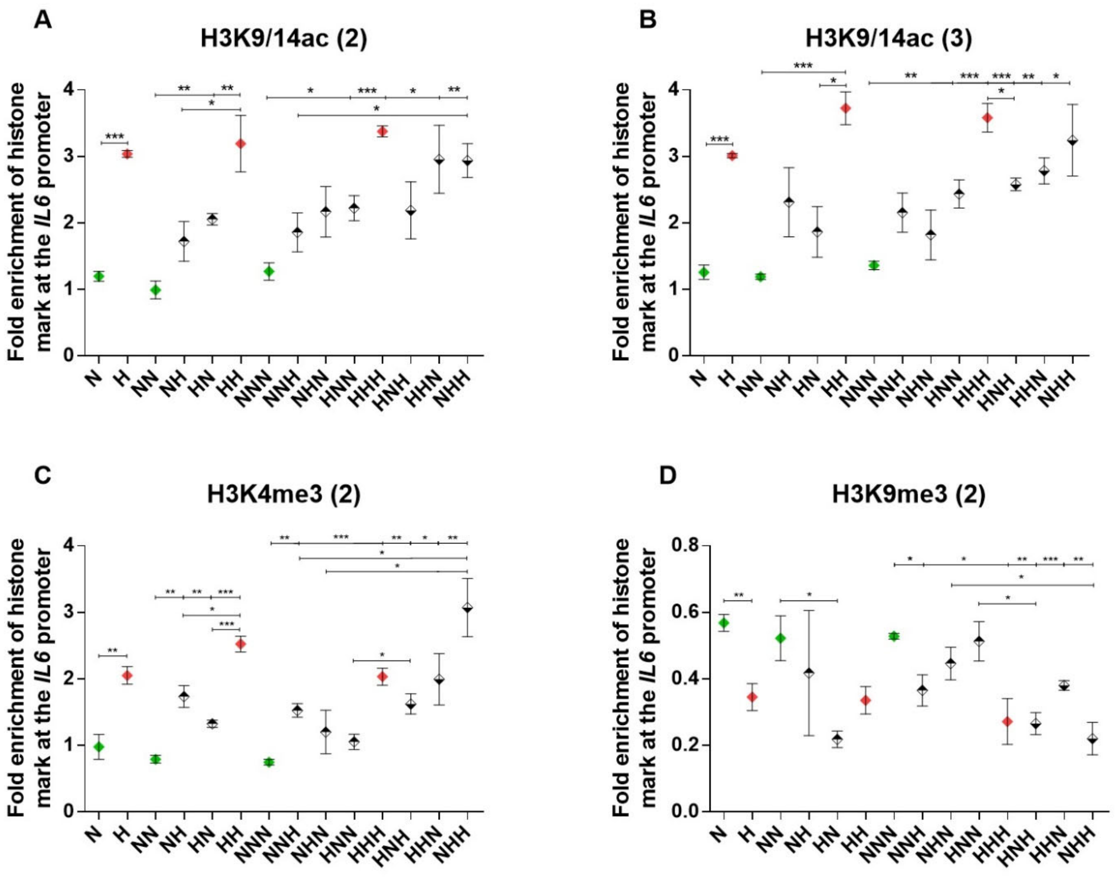

Supplementary figure S1. Fold enrichment of H3K9/K14ac (A, B; Region 2 and 3), H3K4me3 (C), H3K9me3 (D, Region 2) marks at the *IL6* gene promoter (A-C) in studied human visceral preadipocytes (HPA-vs). Variants differentiated chronically in normoglycemic conditions and chronic hyperglycemia (HG) were colored in green and red, respectively. Variants of transient HG treatment applied during one stage of cell culture are marked with rhombuses with a black upper half and variants exposed to HG during two stages are denoted by rhombuses with a black lower half. Data were presented as mean ± SEM. Significant differences were marked by asterisks according to the p value criteria: * - 0.05 ≥ p > 0.01, ** - 0.01 ≥ p > 0.001, *** - p ≤ 0.001. Significant differences estimated in one-way ANOVA with a post-hoc Tukey test were marked by green or red asterisks.

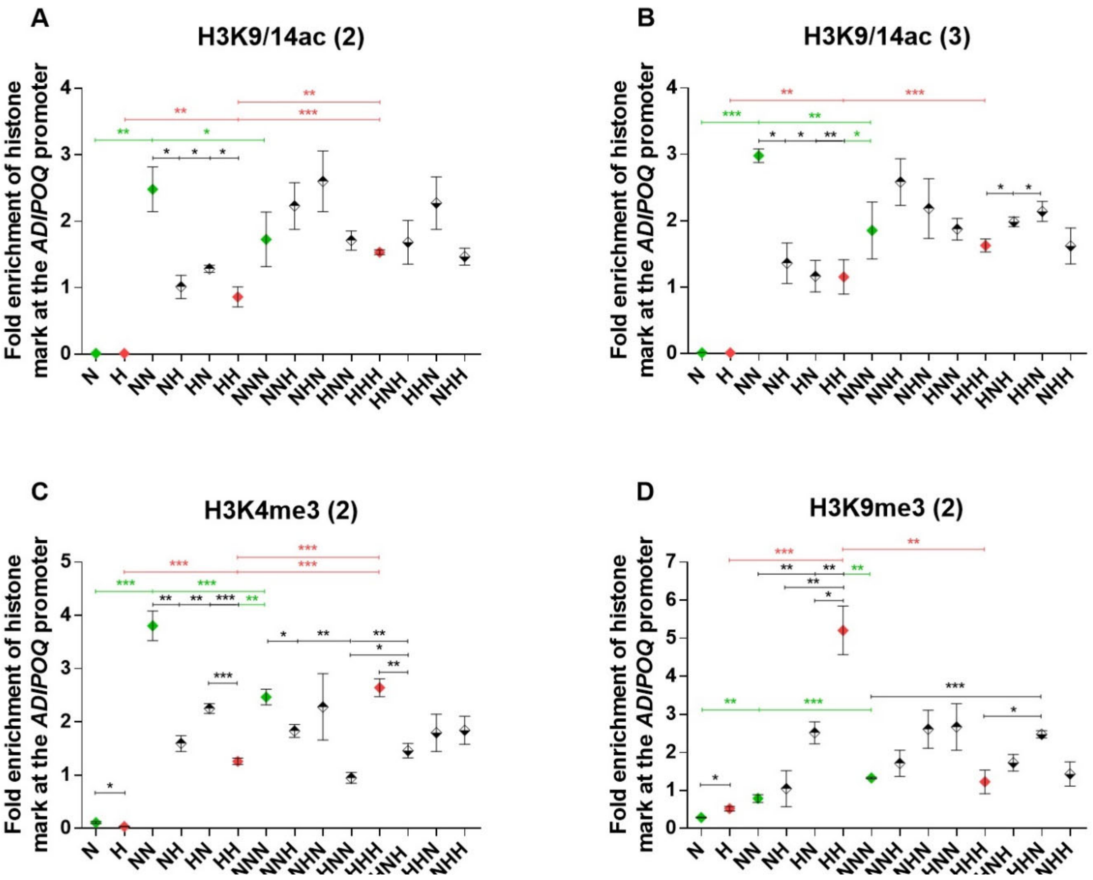

Supplementary figure S2. Fold enrichment of H3K9/K14ac (A, B; Region 2 and 3), H3K4me3 (C), H3K9me3 (D, Region 2) marks at the *ADIPOQ* gene promoter in studied human visceral preadipocytes (HPA-vs). Variants differentiated chronically in normoglycemic conditions and chronic hyperglycemia (HG) were colored in green and red, respectively. Variants of transient HG treatment applied during one stage of cell culture are marked with rhombuses with a black upper half and variants exposed to HG during two stages are denoted by rhombuses with a black lower half. Data were presented as mean ± SEM. Significant differences were marked by asterisks according to the p value criteria: * - $0.05 \geq p > 0.01$, ** - $0.01 \geq p > 0.001$, *** - $p \leq 0.001$. Significant differences estimated in one-way ANOVA with a post-hoc Tukey test were marked by green or red asterisks.
